# Regenerative Medicine Strategies for Spinal Cord Injury: Advances in Stem Cell Therapy

**DOI:** 10.3390/brainsci16050461

**Published:** 2026-04-25

**Authors:** Ahmed I. Anwar, Alaa Abd-Elsayed, Alan D. Kaye

**Affiliations:** 1 School of Medicine, Louisiana State University Health Sciences Center at Shreveport, Shreveport, LA 71103, USA; 2 Department of Anesthesiology, University of Wisconsin, Madison, WI 53706, USA; 3 Department of Anesthesiology, Louisiana State University Health Sciences Center at Shreveport, Shreveport, LA 71103, USA

**Keywords:** spinal cord injury, stem cell therapy, regenerative medicine, neural stem cells

## Abstract

**Highlights:**

**What are the main findings?**

- Stem cell therapies have demonstrated regenerative potential in preclinical models of spinal cord injury
- Clinical studies have reported variable improvements in motor, sensory, and autonomic outcomes

**What are the implications of the main findings?**

- Stem cell therapies may offer therapeutic benefit, but clinical efficacy remains uncertain due to heterogenous outcomes
- Further standardized clinical studies are needed to determine the safety and effectiveness of stem cell therapies for spinal cord injury

**Abstract:**

Spinal cord injuries disrupt the motor, sensory, and autonomic functions routinely carried out by the spinal cord, with injury progressing from primary mechanical damage from the initial trauma to a secondary phase driven by inflammation and cellular cascades. This disruption significantly impacts the patient’s ability to perform basic physiological and voluntary functions seen in a normal spinal cord, which often results in long-term disability and dependence on supportive care. Stem cell therapies, including mesenchymal stem cells, neural stem cells, and induced pluripotent stem cells, have been investigated as potential regenerative approaches that may promote repair through neuroprotection, remyelination, and axonal regeneration. Preclinical studies have demonstrated encouraging results in motor and sensory recovery following injury; however, clinical evidence remains limited and variable. Some studies report improvements in motor and sensory function post-injury, with improvements in bladder and bowel management, tissue repair, and other functions. Overall, the outcomes vary based on cell type, delivery method, and the stage of spinal cord injury. The key challenges of stem cell therapy include safety concerns and the limited number of small-scale studies currently available. Additionally, understanding the variability in therapeutic outcomes and identifying optimal treatment conditions are critical steps toward advancing stem cell therapies in spinal cord injury repair. This review aims to characterize and summarize the stem cell approach to the treatment of spinal cord injuries, while also critically highlighting the limitations of current preclinical and clinical evidence, as well as the importance of continued investigation into the long-term and functional recovery processes and possibilities, as well as the patient’s quality of life following treatment with stem cells for spinal cord injury.

## 1. Introduction

Spinal cord injury is a neurological condition resulting from traumatic or nontraumatic causes that disrupts motor, sensory, and autonomic functions [1]. This disruption can manifest in a wide range of clinical presentations that are seen depending on the level and severity of the spinal cord injury, which can range from a partial impairment to a complete loss of function below the level of the spinal cord lesion. In the United States, there are approximately 55 new cases per million population, representing the annual incidence excluding deaths before arrival at the hospital [2]. This incidence level highlights the significant public health burden associated with spinal cord injuries. Injury to the spinal cord progresses through an immediate phase of physical damage, followed by a secondary phase characterized by inflammation, immune responses, glial and other immune cell activation, and vascular disruption [3]. This combination of effects leads to cellular dysfunction and further tissue damage. The progression from primary to secondary injury also emphasizes the importance of early intervention once a spinal cord injury occurs. For patients presenting with spinal cord injury, imaging using CT and MRIs is important for evaluating the integrity and injury extent [4]. These imaging modalities assist in diagnosis and characterization of injury severity and support treatment planning, allowing clinicians and healthcare teams to monitor the progression or the resolution of the injury over an extended period of time, which makes them an essential tool in the acute and chronic management of a spinal cord injury. This ongoing monitoring is critical for adapting treatment strategies as the patient’s condition evolves following an injury.

Treatment for a spinal cord injury is dependent on the extent of damage and injury. Current treatment focuses on pharmacological management and surgical intervention if needed, with early spinal decompression within the first 24 h [5]. This is considered important for improving neurological recovery. Timely intervention can help minimize secondary injury and also preserve the remaining neural tissue. Additionally, traditional treatments such as immobilization and rehabilitation are used, again depending on the severity of the injury. A potential emerging therapeutic approach under investigation for spinal cord injury includes stem cell therapies, with supportive approaches that use mesenchymal stem cells to support tissue repair and modulation of injury-related processes [1]. Rehabilitation strategies, which include physical and occupational therapy, also remain and continue to be essential components of care, and they aim to maximize the remaining functionality that is available for these patients and also aim to prevent secondary complications that can occur, such as muscle atrophy and contractures, which can occur following a traumatic spinal cord injury. These supportive therapies also complement emerging treatments by enhancing the patient’s overall recovery.

Stem cell-based strategies for spinal cord repair offer a potential regenerative approach under investigation, involving either transplanting exogenous stem cells or endogenously activating neural stem cells within the existing spinal cord to support tissue repair and functional recovery processes [6]. These approaches also provide flexibility in treatment design, depending on patient-specific factors. These stem cell therapies support recovery through multiple mechanisms including replacement of lost or damaged cells (e.g., oligodendrocytes), support for remyelinating, modulation of inflammatory responses, and paracrine secretion of trophic factors involved in tissue repair. Stem cell approaches remain ongoing, and there is a focus on improving their efficacy and safety for clinical practice. Additionally, these therapies may contribute to creating a more permissive microenvironment for regeneration by modulating the inhibitory post-injury environment, including glial scar formation and inhibitory signaling molecules associated with spinal cord injury. Overcoming these inhibitory factors is considered a key challenge in achieving meaningful regeneration in the central nervous system.

Despite the advances in surgical and supportive management for spinal cord injuries, the current available therapies remain largely limited to preventing further damage but not restoring lost neurological function or improving neurological recovery to a meaningful extent. This limitation emphasizes the need for regenerative approaches rather than merely neuroprotective strategies. As a result, there is a continued need for innovative approaches that promote the regeneration of spinal cord tissue. This paper aims to review the roles of stem cell-based therapies in the treatment and repair of spinal cord injuries, with a focus on mechanisms, therapeutic potential, and current challenges in stem cell-based treatments. Overall, by synthesizing current findings in stem cell research and spinal cord injury, this review aims to provide a clear and concise understanding of how these therapies may be optimized and further developed for future potential clinical application in patients with spinal cord injury. Optimization of this kind is crucial for future translation toward clinical use.

## 2. Methodology

This narrative review was conducted using a targeted literature search of the PubMed database. Articles were identified using combinations of relevant keywords, including “spinal cord injury”, “stem cell therapy”, “regenerative medicine”, “neural regeneration”, and “cell-based therapies”. No strict date restrictions were applied, allowing for the inclusion of both the foundational and recent studies to provide a comprehensive overview of the field. Studies were selected based on their relevance to the pathophysiology of spinal cord injury, the therapeutic potential of stem cell-based interventions, and translational or clinical considerations. Priority was given to peer-reviewed research articles, systematic reviews, and key preclinical and clinical studies that significantly contributed to understanding mechanisms of injury, therapeutic strategies, or clinical outcomes.

## 3. Pathophysiology of Spinal Cord Injury

**Figure 1:**
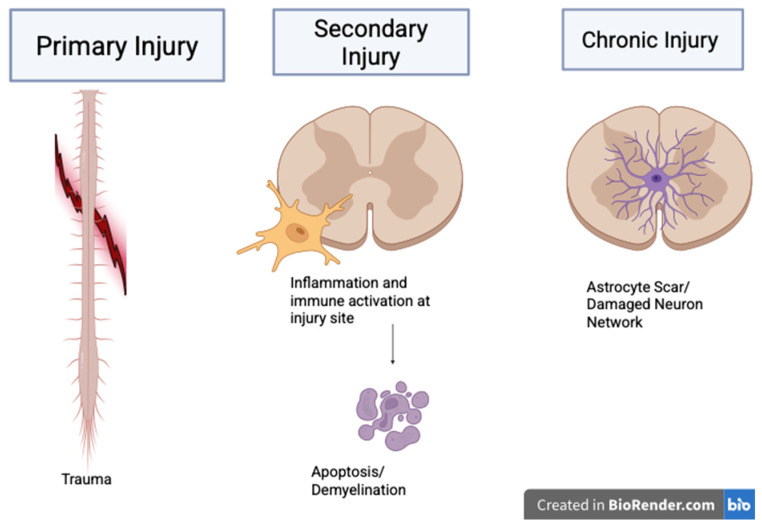
Illustrates the temporal and mechanistic progression of spinal cord injury (SCI). The primary injury phase involves immediate mechanical trauma to the spinal cord, resulting in structural disruption of neural tissue. The secondary injury phase follows, characterized by activation of microglia and immune cells, leading to inflammatory responses, apoptosis, and demyelination that further exacerbate tissue damage. The chronic phase is marked by glial scar formation mediated by astrocyte activation and persistent disruption of neuronal networks, contributing to long-term functional deficits. The figure highlights the sequential evolution of SCI pathology from acute mechanical damage to chronic inhibitory repair conditions. Created in https://BioRender.com.

Spinal cord injury pathophysiology is complex, multifactorial, and progressive after injury, as it involves the disruption of the normal interactions and interplay among neurons, astrocytes, oligodendrocytes, and other neural and glial cell populations, which impairs the synchronized and coordinated spinal cord function required for normal neurological function [7,8]. This disruption of cellular interactions leads to dysfunction that is observed following an injury and usually extends beyond the initial injury site. Spinal cord injury is considered to occur in two phases: primary and secondary injury (Figure 1), as illustrated in a model of injury progression. The primary phase occurs the moment the trauma occurs, while the secondary phase occurs and evolves over multiple hours to weeks after the event, which is the phase in which extensive tissue damage occurs following traumatic injury [8,9]. A delayed progression also provides a therapeutic window for intervention as summarized in Figure 1.

Primary injury includes mechanical forces such as hyperflexion, rotation, and other trauma, which lead to immediate structural damage, which can include hemorrhage, edema, and axonal damage [9]. The initial trauma also causes necrotic cell death of neurons, glial cells, and structures, as well as of the vascular system in the area, leading to ischemia and oxygen depletion at the lesion site. The secondary injury includes an amplification of the initial trauma through a cascade of biochemical and cellular processes that are temporarily and mechanistically distinct from the primary phase. These include inflammation, apoptosis, demyelination, and excitotoxicity [8,10]. Inflammatory responses include the activation of microglia and other immune cells, such as neutrophils, and, overall, this increases the tissue damage that primarily occurs [10]. This is a widely accepted biphasic model that also serves as a foundation for understanding therapeutic interventions aimed at limiting secondary damage following a spinal cord injury and promoting recovery of remaining cells and functionality (Figure 1). These processes, overall, create a hostile microenvironment for repair and regeneration, further limiting the potential for spontaneous recovery and regeneration following a spinal cord injury.

The loss of oligodendrocytes can lead to demyelination and impaired nerve signal conduction, thereby decreasing the prognosis for functional recovery [8]. This impairment of the signal condition also affects the efficiency and speed of neural impulse transmission, further contributing to the functional deficits observed in patients with this injury. The environment in the spinal cord injury area is very unfavorable for regeneration due to a lack of support and inhibitory molecules. The molecular disturbances also lead to dysfunction, resulting in long-term symptoms and deficits, including motor and sensory deficits. Additionally, spinal cord injuries extend beyond the spinal canal, with injuries potentially affecting system functions, which complicates recovery [8,11]. This systemic impact seen here highlights the need for a comprehensive treatment strategy that can address the neurological and physiological complications associated with spinal cord injuries to promote better repair for patients following the injury (Figure 1).

## 4. Stem Cell-Based Regenerative Strategies in Spinal Cord Injury

Stem cells and glial cells are currently being explored for their role in neuroprotection, immune modulation, angiogenesis, and formation of new functional central nervous system (CNS) circuits [12]. These mechanisms have primarily been demonstrated in preclinical SCI models, with limited and heterogenous clinical translation to date. These roles are essential in both protecting existing neural structures and facilitating the formation of new pathways necessary for neural recovery. Biomaterials also serve as scaffolds for tissue repair and vehicles for cells, growth factors, and drugs [13]. These scaffolds also provide structural support to help guide cellular growth and organization within a specific damaged area. Electrical stimulation is also being investigated to promote plasticity, potentially improving outcomes for regenerative medicine approaches [14]. However, most evidence for biomaterials and electrical stimulation in SCI remains preclinical or early-phase translational. This stimulation may also enhance neural tissue responsiveness and improve the integration of regenerative therapies for spinal cord injury. Stem cells also release regenerative factors that promote neuronal survival and immune modulation, demonstrating their role in treating spinal cord injuries [15]. These paracrine effects are most consistently supported by mesenchymal stem cell-based preclinical studies. These combined approaches suggest that a multifaceted treatment strategy can be, and possibly is, more effective than a single modality alone, as it can address multiple aspects of injury and recovery simultaneously, thereby improving the patient’s quality of life following a spinal cord injury. However, clinical evidence directly comparing combination strategies remains limited. Additionally, by targeting multiple pathways, it also produces more consistent and sustained improvements in function in preclinical studies, though this has not yet been consistently validated in large clinical trials.

### 4.1. Stem Cell Therapy

Stem cell therapy is a promising approach for spinal cord injury, aiming to restore neural tissue, modulate inflammation, promote axonal regeneration, and improve functional outcomes for patients with an injury. This multifaceted approach aligns with the nature of spinal cord injury by emphasizing the need to understand the structural and functional deficits and to repair them to regain spinal cord function for the patient. Multiple cell types, delivery strategies, and combination approaches are being explored in both preclinical and clinical studies. The complex diversity of these approaches reflects the complexity of spinal cord injuries and the need for there to be a tailored treatment to individual patient conditions and individual patient differences and injury characteristics, depending on their type and extent of spinal cord injury. Individualized treatment strategies can also help maximize therapeutic effectiveness across diverse patient populations.

Importantly, evidence supporting these different approaches varies depending on the type of study, whether it is a preclinical animal study or human clinical trial, and outcomes are influenced by injury severity, timing, and cell source, which are all critical factors in interpreting the efficacy of treatment. A summary of major cell-based regenerative strategies is provided in Table 1.

### 4.2. Mesenchymal Stem Cells (MSC)

Mesenchymal stem cells (MSCs), including bone marrow-derived (BM-MSCs), umbilical cord-derived (UCMSCs), and Wharton’s jelly MSCs (WJ-MSCs), are multipotent and exhibit low immunogenicity [18,19]. The low immunogenicity also makes it suitable for transplantation because of the lower risk of rejection and because it improves prognosis following transplantation. The therapeutic effects of these mesenchymal stem cells arise from the secretion of neurotrophic and angiogenic factors, including VEGF and GDNF, modulation of inflammation, and reduction in astroglial scarring, which helps restore the blood-spinal cord barrier integrity and decrease the negative functional disabilities following a spinal cord injury [12,13,16,17]. Notably, the strongest evidence for this mechanism derives from preclinical spinal cord injury models [16]. However, these effects are primarily supported by preclinical models, with clinical trials showing more variability depending on injury stage, delivery route, and dosing strategy.

Genetic modifications and the co-transplantation with Schwann cells or olfactory ensheathing cells (OEC) also enhance the efficacy of stem cell therapy for spinal cord injury [31]. Such enhancements can increase regenerative potential, thereby improving the success of the therapy. Clinical trials have shown improvements in motor and sensory scores, bladder management, and the conclusion that single-cell therapies for chronic injuries are less effective than multiple-cell therapies [18,19,21]. However, reported clinical outcomes are inconsistent across studies, and differences in follow-up duration. These findings emphasize the importance of optimizing dosage, timing, and combinations of strategies to maximize the therapeutic benefit of stem cell-based repair and regeneration following spinal cord injury. Optimizing the variables shown here is essential for achieving consistent, reproducible outcomes.

### 4.3. Neural Stem and Progenitor Cells (NSCs)

Neural stem and progenitor cells (NSCs) can differentiate into neurons, astrocytes, and oligodendrocytes, all of which support remyelination and repair of synapses following spinal cord injuries and trauma [32]. This capacity for differentiation allows them to replace damaged or lost cells within the spinal cord directly. Recent translational work has led to the initiation of early clinical trials using hiPSC-derived neural stem/progenitor cells, particularly in subacute spinal cord injury, which demonstrates the emerging clinical potential [20]. Most evidence for NSCs is derived from preclinical studies, with early-phase clinical studies remaining limited.

NSC transplantation has sensory and motor improvements following therapy and exhibits long-term safety, with adverse events related to the procedure rather than the actual implantation of the stem cells themselves [19,21]. In a phase one clinical trial of human spinal cord-derived neural stem cells, all participants tolerated transplantation well, and long-term follow-up demonstrated durable evidence of neurological improvement with sustained gains in motor and sensory scores in patients up to five years post transplantation [22]. These outcomes remain variable depending on injury completeness and timing of intervention, highlighting the need for standardized reporting across studies. This suggests that the therapy is well-tolerated when administered correctly.

### 4.4. Induced Pluripotent Stem Cells (iPSCs)

Induced pluripotent stem cells (iPSCs) offer an alternative to embryonic stem cells by reprogramming adult somatic cells. Preclinical studies in this stem cell field have indicated that iPSC-derived neural cells can replace lost neurons, promote axonal growth, and provide support [23]. This provides a flexible, patient-specific approach to therapy depending on the extent and type of injury. Current evidence is largely preclinical, and clinical translation remains limited [24].

However, iPSCs also carry tumor risks and may acquire the genetic or epigenetic abnormalities required for tumor growth. This causes a clinical safety issue and risks [25,26]. These limitations significantly restrict current clinical application and necessitate long-term safety studies. Additionally, careful monitoring is required to mitigate the risks.

### 4.5. Schwann Cells and Olfactory Ensheathing Cells (OECs)

The supportive cells of the nervous system, including Schwann cells and OECs, play roles in axonal guidance, remyelination, and regeneration [27,28]. Clinical studies have reported partial recovery of motor and sensory function following transplantation, with some evidence of greater improvement in studies using mesenchymal stem cells or combined cell-based approaches [29]. Transplantation of Schwann cells and OECs has also shown very high improvement post-transplantation and has surpassed expected spontaneous recovery in chronic spinal cord injuries [19,30]. These findings are primarily based on small-scale clinical studies and require validation in larger controlled trials. Additionally, Schwann cell transplantation has played a role in decreasing the pro-inflammatory innate response following spinal cord injury, potentially improving the environment for repair [33]. These results suggest that combining multiple cell types may enhance overall therapeutic outcomes by leveraging the complementary mechanisms of action of the different modalities. Synergistic approaches may provide a more comprehensive regenerative effect, improving recovery following an injury.

Overall, stem cell therapies for spinal cord injury have demonstrated great potential, but there is still significant variability in cell type, injury stage, and delivery method, which limits direct comparisons between patients. This variability, as seen here, also complicates the establishment of standardized treatment protocols. A combination of strategies and rehabilitation is critical to maximize the recovery of patients receiving stem cell therapy for spinal cord injuries [18,19,21,25,30]. Standardization of protocols and outcome measures will be essential in the future for comparing results across studies and advancing clinical translation in practice. Consistent methodologies will also allow for more accurate evaluation of treatment effectiveness.

## 5. Clinical Translation of Stem Cell Therapy in Spinal Cord Injury

Stem cell therapy for spinal cord injury has been increasingly used and translated from experimental and preclinical models into human clinical studies [34,35,36,37,38]. However, the majority of supporting evidence remains derived from early-phase or small-scale clinical trials with heterogenous study designs (Table 2). Clinical studies have shown that implementation in both acute and chronic spinal cord injury is reported in early clinical studies, but outcomes vary significantly depending on injury severity, timing of intervention, and study design [34,35,36,39]. The use of autologous stem cells, including mesenchymal and bone marrow stem cells, supports its applicability by reducing immune rejection during transplantation [35,37,38]. The clinical translation of stem cell therapy for the repair and regeneration of neural tissue involves combining stem cells with biomaterials or surgical interventions, both of which are used to improve outcomes following transplantation [34,37]. However, clinical evidence supporting combination approaches remains limited and largely preclinical. Examples of these procedures include peripheral nerve grafts, scaffolds, and transplantation of mesenchymal stem cells [34,40].

Additionally, scaffolds and bone marrow mononuclear cells are also being investigated as adjunctive strategies in stem cell-based spinal cord injury repair [37]. The approach here aims to enhance cell survival and integration of the transplanted stem cells, although evidence supporting these benefits in human studies remains limited. Biomaterial scaffolds also may improve stem cell survival and support axonal regeneration by providing structural guidance across the lesion site, while creating a controlled environment [41]. This helps with structural support and the regeneration of neural tissue and the surrounding area. There are complications, such as biomaterial degradation, however, which is a translational challenge seen with stem cell therapy [34].

Clinical studies have reported improvements in motor and sensory function in patients who receive stem cell therapy; however, the results vary depending on the cell type used, injury chronicity, and delivery method [34,36,38]. The observed benefits of stem cell therapy in patients include improvements in ASIA scores and neurological levels [34]. Increasing motor and sensory recovery rates are also seen in patients [36]. Additionally, enhanced bladder and urologic function are observed, and daily functioning also improved following stem cell therapy [36,38]. Additionally, some clinical studies have shown that stem cell therapy is more effective than doing just rehabilitation, highlighting the potential for this new therapy in spinal cord injuries.

Despite promising outcomes in clinical studies, efficacy remains inconsistent across multiple studies [35,37]. The limited improvement is seen in some cases, with a limited amount of motor recovery and partial sensory improvement following stem cell therapy [35]. In some studies, there is also no significant motor recovery despite structural repair [37]. These mixed outcomes suggest that anatomical improvement does not always translate into meaningful functional recovery. This highlights the complex, multifactorial pathways and systems involved in spinal cord injury and rehabilitation. There are multiple injury stages, including acute and chronic, and the treatment protocol and patient-specific factors all influence and guide treatment and favor favorable outcomes or unfavorable outcomes seen with stem cell therapy [35,37].

Stem cell therapies are also considered generally well tolerated in early clinical studies with no life-threatening adverse effects reported in multiple clinical studies [36,37]. There are minor complications, such as post-operative seroma formation, which is due to scaffold degradation [34]. Overall, this lack of serious side effects supports that, clinically, stem cell therapy has demonstrated an acceptable short-term safety profile in selected study populations.

There is also evidence that the timing of the intervention depends on whether the patient is in the acute or chronic injury stage, with earlier intervention often associated with improved neurological recovery compared to delayed treatment [42]. That appropriately timed therapy is important for favorable outcomes [35,37]. Evidence suggests that increasing the dose and doing nothing else does not necessarily improve patient outcomes, and that repeated administrations of the stem cell therapy may actually enhance the neurological recovery of patients [36]. Additionally, clinical studies have demonstrated that while neurological improvements are observed, they do not always translate into independent movement or a full functional recovery [34,37]. This distinction highlights that improvement in clinical scores may not correspond to functional independence. This again highlights that while stem cell therapy is promising, in clinical scenarios, it is not a guaranteed fix for the injury following spinal cord trauma. Patients have shown motor gains while receiving this therapy but are unable to walk independently [34]. This highlights that there is also a spectrum of improvement following this therapy.

Overall, stem cell therapy in clinical practice has shown potential therapeutic benefit, but successful clinical translation still requires standardized protocols and optimization of patient criteria for selecting the right patients.

## 6. Current Challenges

Key limitations of stem cell therapies include safety concerns, such as immune reactions, as well as poor cell survival and limited integration into the damaged tissue [12]. These limitations, as seen here, reduce the overall effectiveness of the treatment and present barriers to the widespread clinical translation of stem cell therapy for spinal cord injuries. Additionally, clinical translation is challenged by variability in cell sources and protocols, an incomplete understanding of underlying mechanisms, and inconsistent results from the small studies currently available. The use of immunosuppressive drugs to support transplanted cell survival may improve early recovery, but does not enhance long-term outcomes, highlighting the unresolved trade-off in clinical translation [43]. Additionally, variability in delivery routes contributes to translational inconsistently, despite evidence of stem cell migration to the injury site, alongside reported risks such as vascular embolism [44]. This inconsistency makes it difficult to conclude the efficacy of this treatment at present. Additionally, challenges include limited stem cell sources, tumor risks, and poor survival in the environment of spinal cord injury, which complicates and decreases effective transplantation [45]. The clinical trials also include small patient numbers, short follow-ups, and variable methods, which reduce the ability to standardize and generalize results from stem cell therapy in spinal cord injuries. Addressing these challenges will require multiple large-scale, well-designed clinical trials, which will be difficult; however, improved methods for long-term tracking of safety and efficacy outcomes will ensure that stem cell therapy is a potentially viable and safe investigational option for patients with spinal cord injuries. An improved study design and data collection will also be critical in validating these therapies.

## 7. Future Directions

Future strategies may also increase the use of biomaterials, scaffolds, and constructs to enhance the delivery of the stem cells and the organization and integration of stem cells within the injured spinal cord [46,47]. These platforms aim to replicate the native microenvironment and improve regenerative outcomes. Technologies such as CRISPR-Cas9 and targeted genetic modifications are also expected to play a unique role in enhancing stem cell therapeutic potential by promoting expression of neurotrophic factors, which improves cell survival and also allows for precise control over the regenerative pathways [48].

Induced pluripotent stem cells also provide a foundation for individual therapies derived from the patient’s own somatic cells. Future directions within this specific therapy include refining patient-specific differentiation protocols and tailoring treatments that are based on individual biological variability to improve therapeutic precision [47]. Preconditioning of stem cells using hypoxia and growth factors is also an emerging new approach that improves the resilience of the stem cells and their regenerative capacity prior to transplantation into the injured region. These strategies aim to increase the survival and functional performance of the transplanted stem cells in the injured spinal cord environment [48]. Additionally, future directions with combination therapy with spinal cord stimulation represent a strategy with the potential to restore functional connectivity and enhance motor recovery beyond structural repair alone [48].

## 8. Conclusions

Spinal cord injury remains a major clinical challenge, with limited therapies capable of restoring the lost neurological function. Preclinical evidence supports stem cell-based regenerative strategies having the ability to promote neuroprotection, remyelination, axonal repair, and modulation of the post-injury inflammatory environment, while early clinical studies suggest that these approaches are feasible and may provide modest improvement. However, clinical efficacy remains uncertain due to the heterogeneity of cell type, delivery method, and injury stage. Safety concerns, including immune reactions, tumorigenic risk, and poor graft survival, also continue to limit the translation of stem cell therapy. Future progress may require standardized clinical protocols, rigorous controlled trials, long-term safety monitoring, and improved patient stratification. Until these challenges are addressed, stem cell therapy for spinal cord injury should remain an investigational rather than established clinical treatment option.

## Figures and Tables

**Table 1:** Overview of stem cell and glial cell-based regenerative therapies for spinal cord injury, including mechanism, reported outcomes, and key limitations.

Cell Type	Mechanism	Outcomes + Limitations
MSC	Neurotrophic/angiogenic signaling, immunomodulation, reduced astroglia scarring, BBB restoration [16,17]	Motor/sensory improvement and AIS score gains reported, but outcomes vary with injury stage, dose, and delivery route [17,18,19]
NSC	Differentiate into neurons, astrocytes, oligodendrocytes, remyelination, and synaptic repair [20]	Motor/Sensory improvement and long-term safety; phase 1 trial shows durable gain up to 5 years post-transplant, but evidence remains limited and heterogenous [19,21,22]
iPSC	Neuronal replacement and axonal growth support in preclinical models [23,24]	Preclinical functional improvement, limited clinical translation with safety concerns including tumorigenicity [25,26]
Schwann/OEC	Axonal guidance, remyelination, regenerative support [27,28]	Partial motor/sensory recovery; improved outcomes in combination therapies, mostly small clinical studies [19,29,30]

Caption: This table summarizes major cell-based regenerative strategies investigated for spinal cord injury, highlights mechanisms of action and reports outcomes.

**Table 2:** Clinical Translation of Stem Cell Therapy in Spinal Cord Injury.

Category	Summary
Clinical Evidence & Variability	Early clinical translation with heterogenous outcomes influenced by stage of injury, timing, and severity [34,35,36,37,38,39]
Interventions & Approaches	Autologous MSCs and bone marrow cells, combined with scaffolds or surgical techniques to enhance repair [35,37,38,40,41]
Outcomes, Safety, & Limitations	Motor/sensory and functional improvements reported but inconsistent; generally safe with minor complications; limited functional independence despite some neurological gains [34,35,36,37,38]
Modulating Factors	Timing (acute vs. chronic) and repeated dosing can influence recovery outcomes [36,42]

Caption: Summary of clinical translation highlighting characteristics, intervention strategies, reported outcomes, and influencing factors.

## Data Availability

No new data were created or analyzed in this study.
